# On the Treatment of the More Common Forms of Skin Diseases Met with in Edinburgh

**Published:** 1855-08

**Authors:** John Hughes Bennett

**Affiliations:** Professor of the Institutes of Medicine, and of Clinical Medicine, in the University of Edinburgh


*On the Treatment of the more Common Forms of Skin Diseases met with in Edinburgh.* By JOHN HUGHES BENNETT, M. D., F. R. S. E., Professor of the Institutes of Medicine, and of Clinical Medicine, in the University of Edinburgh.

Since the addition of a ward for skin diseases to the clinical department of the Royal Infirmary, I have had ample opportunities of determining what are the more common forms of cutaneous eruption met with in this city, and of trying various modes of treatment. A short account of the results of my experience in this department, excluding the eruptive fevers, may not be unacceptable to my medical brethren.

*Eczema* is by far the most common disease met with, both in its acute and chronic forms. The local treatment I have found most efficacious is that which I recommended, in the August number of the Journal for 1849. It consists in keeping the affected part moist with lint or linen saturated in a very weak alkaline solution, consisting of soda sub carb. ½ drachms to a point of water. For this purpose it is necessary to cover the moistened lint with oil silk, or gutta percha sheeting, which should well overlap the lint below, so as to prevent evaporation. The usual effect is soon to remove all local irritation, and especially the itching or smarting so distressing to the patient; to keep the surface clean and prevent the accumulation of those scabs and crusts, which in themselves often tend to keep up the disease. After a time, even the indurated parts begin to soften, the margins of the eruption lose their fiery red color, and merge into that of the healthy skin, and finally the whole surface assumes its normal character.

In private practice it is often a matter of great difficulty to secure a proper application of the lotion. Individuals are slow to accept the idea that constant moisture of the part is absolutely necessary for the treatment, and hence vigilant superintendence and frequent visits are requisite, in order to watch the progress of the case. Even in the hospital constant watchfulness is necessary, to see that the nurses properly cover the eruption; and when, as sometimes happen, this task is given to the patients themselves, it almost always fails. Then there are some portions of the surface which it is very difficult to keep moist and well covered, such as the face and axillæ. But, by carefully adapting lint and gutta percha sheeting, attaching strings to the edges of the latter, so as to keep the whole in its place, I have never failed in ultimately carrying out my object.

In addition to stating what I have found to be useful, it is important to say what I have, on careful trial, ascertained to be useless or injurious. Perhaps no remedy is more generally employed in this and a variety of other skin diseases than citrine ointment, an application that I have always found to irritate and make eczematous eruptions worse. At the same time there are some very chronic forms of the disease, which I have been told are cured by this preparation. but what these are I have been unable to ascertain. Indeed, all greasy applications whatever, in the majority of cases, are useless, and the patients themselves inform me, are very "heating." In some rebellious chronic instances, I have thought the oil of cade has been beneficial, applied locally, although I have not yet tried it sufficiently often to recommend it strongly. In a few cases of acute eczema, I have tried the freezing process recommended by Dr. Arnott, but the salt of the frigorific mixture, and the cold itself, has caused apparently so much agony that I have been deterred from using it, especially when the emollient moist alkaline application is so efficacious. This mode of treatment, however, undoubtedly demands further trial, and I propose to report a more extended experience of it on some future occasion.

*Herpes.*—This disease generally runs its course in about fourteen days, and requires no treatment whatever, further than an acetate of lead lotion to allay the smarting. It is not very common.

*Scabies* occurs very frequently, and is cured by a host of remedies. A strong lather, made of common soft soap and warm water, twice a day, answers very well. The question with scabies is not what remedy is useful, but which will cure it in the shortest period. The most extensive experience at St. Louis has shown that the sulphur and alkaline, or Helmerinch's ointment, cures itch, on an average, in ten days. That sulphur, however, is not the active remedy, I have satisfied myself of by experiment. Soft soap, as we have seen, which contains alkali, and even simple lard, if pains be taken to keep the parts constantly covered with it, will cure the disease as soon as sulphur ointment. I have tried the Stavesacre ointment, recommended by M. Bourguinon, in only a few cases, but found it to answer very well. Its superiority, however, over other applications, I am not yet prepared to admit.

*Pemphibus.*—This is rather a rare disease, and when chronic, coming out in excessive crops, is very rebellious. Two cases which entered the Infirmary last winter were cured in a few weeks by the weak alkaline wash, applied as in the case of eczema, combined with generous diet.

*Impetigo.*—This affection in all its forms is very common, and is best treated by the weak alkaline wash, exactly the same as in eczema. In the chronic forms which attack the shin of men, constituting one of the varieties of mentagra, the same treatment cures the most rebellious cases, if the moisture be constantly preserved. For this purpose the hair must be cautiously cut short with sharp scissors, and the razor carefully avoided. If the side of the cheek covered by the whisker be attacked, removal of the hair thence also is essential to treatment. A bag or covering accurately adapted to the part affected must be made of gutta percha sheeting, and tied on with strings. This may be covered with a piece of black silk, to allow the individual to go about and carry on his usual occupations. In this way I have frequently seen chronic impetigo of the shin, of from eight to ten years' standing, completely removed in a few weeks. But then the surface must be kept *constantly* moist, a circumstance requiring great care and determination on the part of the patient. When it becomes necessary to shave, flour and warm water should be used, and not soap. Alkalies applied from time to time only, as in the wash or soap, always irritate, although, when employed continuously, they are soothing.

*Ecthyma* is not a common disease, and usually presents itself as the E. *cacheticum*, requiring in addition to the alkaline wash locally, a generous diet.

*Acne* is a disease always requiring constitutional rather than local remedies. Although not uncommon in private, it is rare in hospital practice. Careful regulation of the diet, abstinence from wine and stimulating articles of food, watering places, baths, etc., etc., constitute the appropriate treatment.

*Rupia.*—This disease I have never seen occur but in individuals who have been subjected to the influence of mercurial poisoning. Hydriodate of potassium and tonic remedies, with careful avoidance of mercury in all its forms, is the treatment I have found most successful.

*Lichen and Prurigo.*—In both these affections constant inunction with lard is as beneficial as constant moistnre in the eczematous and impetigious disorders. In the prurigo of aged persons, the *Uny. Hyd. Precip. Alb.* is a useful application, although the disease is not unfrequently so rebellious as only to admit of palliation. The chronic papular diseases often constituted the despair of the physician.

*Psoriasis*, and that modification of it known as lepra, are very common diseases, and are uniformly treated by me externally with pitch ointment. I have satisfied myself by careful trials that it is the pitch applied to the part that is the beneficial agents, as I have given pitch pills, and infusion of pitch, largely internally without benefit. With the hope of obtaining a less disagreeable remedy, I have frequently tried creosote, and naptha ointment and washes, but also without benefit. Lastly, I have caused simple lard to be rubbed in for a lengthened time, but without doing the slightest good. The oil of cade is also very useful, especially in psoriasis of the scalp. Internally, I give five drops each of Fowler's solution, and of the tr. cantharidis. It is rare that the internal treatment alone produces any effect on a case of psoriasis of any standing. If a case resists this conjoined external and internal treatment, I have always found it incurable. About a year ago I carefully treated a series of cases internally, with Donovan's solution, without producing the slightest benefit.

*Lupus* is a constitutional disease, and must be treated by cod liver oil, and all those remedies useful for scrofula, of which it is a local manifestation. The external treatment is surgical, consisting of the occasional application of caustics, red lotion, ointment, etc., according to the appearance of the sore.

*Fevus* is a very common disease in Edinburgh, and is most readily removed, first, by poulticing the crusts till they fall off, and the skin presents a smooth, clean surface; secondly, by shaving the hair; and, thirdly, by keeping the scalp continually covered with oil, so as to exclude the atmosphere, and prevent the growth of the parasitic fungi, which constitutes the disease. A continuance of this treatment for six weeks, produces a cure in young persons, if combined with cod liver oil, generous diet, and anti-scrofulous remedies internally. I have tried the lotion of sulphurous acid, recommended by Dr. Jenner, and found it successful in a few cases, but the treatment by oil is so easy as to be far preferable to it. Very chronic cases are cured with difficulty, but so long as the oil is applied, the disease never returns, and mere freedom from the disgusting crusts is a great gain.

*Scalp Diseases* must be treated according as it depends on eczema, impetigo, psoriasis, or favus, in all cases first removing the crusts with poultices, then keeping the head shaved, and, lastly, applying alkaline washes, pitch ointment, or oil, according to the directions formerly given. Ringworm is a disease I have never seen in Edinburgh, and of what it consists, I am ignorant. Some writers apparently consider it to be favus, and others a form of herpes. On two or three occasions I have seen a scaly disease of the scalp, in the form of a ring—that is lepra, which I have cured by pitch oitment, or oil of cade. My friends, Dr. Andrew Wood, informed me some time ago, that he banished it from the Heriot's Hospital school by condensing on the eruption the fumes of coarse brown paper, and thus causing an empyreumatic oil, or kind of tar, to fall upon the part. This has led me to suppose that it is a scaly disease, and a form of lepra or psoriasis.

So-called *syphilitic diseases* of the skin, are, in my opinion, the various disorders already alluded to, modified by occurring in individuals who have suffered for periods more or less long, from the poisonous action of mercury. A longer time will be required for their cure, but the same remedies locally, conjoined with hydriodate of potassium, in smaller doses, with bitter infusions, tonics, and a regulated diet, offer the best chance of success.

The great difficulty in the treatment of skin diseases, generally consists in their having been mismanaged in the early stages—a circumstance I attribute to their not having, until a recent period, been much studied by clinical students. Many chronic cases of eczema are continually coming under my notice, which, in their acute forms, have been treated by eitine ointment, or other irritating applications, which almost invariably exasperate the disorder. I shall not easily forget the case of one gentleman, covered all over with acute eczema, who had suffered excessive from its having been mistaken for psoriasis, and rubbed for some time with pitch, ointment. In the same way I have seen a simple herpes, which would have readily got well if left to itself converted into an ulcerative sore, by the use of mercurial ointment. Nothing is more common than to confound chronic eczema of the scalp with favus, although the misroscope furnishes us with the most exact means of diagnosis. I need scarcely say that the correct application of the remedies I have spoken of can be secured by an acurate discrimination, in the first instance, of the diseases to which they are applicable.

The general constitutional treatment in all these cases seldom demands aperient or lowering remedies, except in young and robust individuals with febrile symptoms. In the great majority of cases, cod liver oil, good diet, and tonics are required. In a few instances, sedatives, both locally and internally, are necessary to overcome excessive itching or irritation. These the judicious practitioner will readily understand how to apply according to circumstances.—*Ibd.*

